# How to treat a “sweetheart” in mitochondrial cardiomyopathy

**DOI:** 10.1038/s44321-024-00070-w

**Published:** 2024-05-09

**Authors:** Hsin-Pin Lin, Derek P Narendra

**Affiliations:** Inherited Movement Disorders Unit, Neurogenetics Branch, National Institute of Neurological Disorders and Stroke, National Institutes of Health, Bethesda, MD 20892 USA

**Keywords:** Cardiovascular System, Genetics, Gene Therapy & Genetic Disease, Organelles

## Abstract

D. Narendra and HP. Lin discuss the metabolic therapeutic strategy for the treatment of mitochondrial cardiomyopathies as reported by H. Kawamata and colleagues, in this issue of *EMBO Mol Med*.

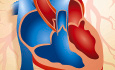

## Teaser

Mitochondria are the powerhouses of the cell, and they are fuel flexible. They can generate power from nearly every abundant carbon source in the body, including carbohydrates, amino acids, and fatty acids. When mitochondria are sick, however, some fuels may be better than others – and the best may not come in “heart healthy” packaging. In this issue of EMBO Molecular Medicine, Southwell et al, report dramatic rescue of a mitochondrial cardiomyopathy by forcing sick mitochondria to use fatty acids, delivered through a high fat diet, rather than sweet glucose they seem to prefer.

The primary model of cardiomyopathy used in the study was previously developed by the authors and results from a dominant disease-causing mutation (p.S59L) in the mitochondrial protein CHCHD10 (Anderson et al, 2019). In humans, mutations in CHCHD10 cause mitochondrial myopathy, including cardiomyopathy, in addition to Amyotrophic Lateral Sclerosis and other motor neuron disorders (Bannwarth et al, 2014; Ajroud-Driss et al, 2015; Shammas et al, 2022). The S59L mutation drives CHCHD10 unfolding into insoluble aggregates within mitochondria, leading to mitochondrial dysfunction and early death from heart failure (Anderson et al, 2019; Genin et al, 2019). These failing hearts prefer glucose to their typical fattier fuel (Sayles et al, 2022).

This observation inspired Southwell et al, to feed their CHCHD10 S59L mice a high-fat diet, reasoning that this intervention might force the sick mitochondria to use fatty acids rather than glucose (Southwell et al, 2024). The intervention worked: the whole-body metabolism of CHCHD10 S59L mice switched from primarily oxidizing glucose to primarily oxidizing fatty acids, as reflected in the measured exchange ratio of O_2_ for CO_2_, which is fuel-specific. The hearts of CHCHD10 S59L mice also adapted by expressing genes facilitating the uptake of fatty acids into the cell and transfer of the acyl chain into the mitochondria via the carnitine shuttle. Strikingly, this change in fuel normalized the heart function of CHCHD10 S59L mice of both sexes and nearly doubled the lifespan of reproductively active females. The mechanism of rescue was even more striking. High-fat diet halved insoluble CHCHD10 in the heart, the main driver of pathogenesis, suggesting it acts as the first step in the pathological cascade. Consistent with protection at an early step, mitochondrial ultrastructure was also preserved by a high-fat diet, and the mitochondrial integrated stress response, which is mediated by the OMA1-DELE1 pathway in response to CHCHD10 S59L accumulation (Shammas et al, 2022), was similarly reduced.

This raised the question: how does a high-fat diet lower levels of toxic CHCHD10 S59L? The authors identified increased turnover of mitochondria, through mitophagy and compensatory mitochondrial biogenesis, as a plausible mechanism. Mitophagy intermediates are known to be increased in the hearts of CHCHD10 S59L mice maintained on a standard diet (Genin et al, 2019). Consistently, Southwell et al, observed increased levels of LC3-II, a protein marker that scales with autophagosome abundance in CHCHD10 S59L mice relative to controls on a standard diet. They additionally observed increased levels of Parkin and PINK1, two proteins that mediate a quality control mitophagy pathway. This increase in mitophagy intermediates is likely a response to decreased mitochondrial damage from toxic CHCHD10. Notably, the high-fat diet dramatically lowered these mitophagy intermediates while also increasing gene expression of transcriptional co-activators that promote mitochondrial biogenesis. As a high-fat diet has previously been shown to stimulate mitophagic flux in the heart, the authors reasoned that this decrease in mitophagy intermediates is most likely due to an increase in mitophagic flux. In this view, mitophagy is stalled in the CHCHD10 S59L mouse hearts, and the high-fat diet provides the need boost to complete this quality control process. As insoluble CHCHD10 is likely a long-lived protein, increasing the overall turnover rate of mitochondria (even in a non-selective manner) would be predicted to promote CHCHD10 clearance. However, the authors left open an alternative explanation. If a high-fat diet somehow lowers CHCHD10 by a yet unidentified mechanism, perhaps there are fewer mitochondrial intermediates simply because there is less mitochondrial damage to clean up. Future work examining mitophagic flux will be needed to differentiate between these alternatives.

Whatever the underlying mechanism, the findings of Southwell et al, bolster the idea that a high-fat diet may be beneficial in at least some forms of mitochondrial myopathy and cardiomyopathy. This idea is not entirely new. The laboratory of Thomas Langer previously reported that conditional knockout of a mitochondrial quality control protease YME1 from the mouse heart causes cardiomyopathy (Wai et al, 2015). The cardiomyopathy in the model was similarly rescued by feeding the mice a high-fat diet, which, as in the CHCHD10 S59L model, increased insulin resistance and decreased glucose utilization by the heart. Similarly, the lab of Anu Suomalainen found that an Atkin’s-like ketogenic diet partially rescues OXPHOS deficiency in a model called the “deletor” mouse, with multiple mtDNA deletions in skeletal muscle from the expression of mutant Twinkle (Ahola-Erkkilä et al, 2010). Ketogenic diets differ from typical high-fat diets, as they have extremely low carbohydrates in addition to the high fat, but the mitochondrial response may be similar. Indeed, as in the CHCHD10 S59L mice, the ketogenic diet promoted mitochondrial biogenesis in the “deletors”, suggesting that increased mitochondrial biogenesis could be a shared protective mechanism in both models.

Notably, the mitochondria in these mouse models, while impaired, have some preserved OXPHOS capacity. This residual OXPHOS activity may be key to their rescue, as a high-fat diet forces cells to rely more heavily on OXPHOS to produce ATP. If the OXPHOS machinery is at least functioning partially, this increased demand can be met by increasing mitochondrial number through mitochondrial biogenesis, as was observed in both the CHCHD10 S59L and “deletor” mice. If the OXPHOS deficiency is severe, however, increased mitochondrial biogenesis may not be able to meet this demand, and with no glucose to fuel glycolysis and no OXPHOS to burn fatty acids, an energy crisis may ensue.

Indeed, this appears to have been the case with the ketogenic diet when it was tested in a small pilot study in patients with mitochondrial myopathy due to mtDNA deletions (Ahola et al, 2016). Some muscle fibers in this form of mitochondrial myopathy have severe OXPHOS deficiency. In response to a ketogenic diet, these fibers underwent energy collapse, resulting in subacute necrotic cell death and lysis. The resulting muscle pain and leak of muscle enzymes led to a premature termination of the clinical trial. Similarly, Southwell et al, found that a high-fat diet, while protecting the CHCHD10 S59L cardiomyopathy model with relatively OXPHOS deficiency, had modest effects in a mouse model with a more severe combined OXPHOS deficiency, due to mutation of frataxin in a model of Fredrich’s ataxia.

It is also unknown how other cell types might respond to a high-fat diet, including the motor neurons in the central nervous system affected in CHCHD10-related ALS. These neurons are metabolically distinct and cannot use directly use fatty acids as fuel, at least not without the lipids first being metabolized by the neighboring astrocytes. Nevertheless, while the high-fat diet may have its limitations, Southwell et al, offer a promising recipe for the treatment of mitochondrial disorders.

## References

[CR1] Ahola S, Auranen M, Isohanni P, Niemisalo S, Urho N, Buzkova J, Velagapudi V, Lundbom N, Hakkarainen A, Muurinen T (2016). Modified Atkins diet induces subacute selective ragged-red-fiber lysis in mitochondrial myopathy patients. EMBO Mol Med.

[CR2] Ahola-Erkkilä S, Carroll CJ, Peltola-Mjösund K, Tulkki V, Mattila I, Seppänen-Laakso T, Oresic M, Tyynismaa H, Suomalainen A (2010). Ketogenic diet slows down mitochondrial myopathy progression in mice. Hum Mol Genet.

[CR3] Ajroud-Driss S, Fecto F, Ajroud K, Lalani I, Calvo SE, Mootha VK, Deng H-X, Siddique N, Tahmoush AJ, Heiman-Patterson TD (2015). Mutation in the novel nuclear-encoded mitochondrial protein CHCHD10 in a family with autosomal dominant mitochondrial myopathy. Neurogenetics.

[CR4] Anderson CJ, Bredvik K, Burstein SR, Davis C, Meadows SM, Dash J, Case L, Milner TA, Kawamata H, Zuberi A (2019). ALS/FTD mutant CHCHD10 mice reveal a tissue-specific toxic gain-of-function and mitochondrial stress response. Acta Neuropathol.

[CR5] Bannwarth S, Ait-El-Mkadem S, Chaussenot A, Genin EC, Lacas-Gervais S, Fragaki K, Berg-Alonso L, Kageyama Y, Serre V, Moore DG (2014). A mitochondrial origin for frontotemporal dementia and amyotrophic lateral sclerosis through CHCHD10 involvement. Brain.

[CR6] Genin EC, Madji Hounoum B, Bannwarth S, Fragaki K, Lacas-Gervais S, Mauri-Crouzet A, Lespinasse F, Neveu J, Ropert B, Augé G (2019). Mitochondrial defect in muscle precedes neuromuscular junction degeneration and motor neuron death in CHCHD10S59L/+ mouse. Acta Neuropathol.

[CR7] Sayles NM, Southwell N, McAvoy K, Kim K, Pesini A, Anderson CJ, Quinzii C, Cloonan S, Kawamata H, Manfredi G (2022). Mutant CHCHD10 causes an extensive metabolic rewiring that precedes OXPHOS dysfunction in a murine model of mitochondrial cardiomyopathy. Cell Rep.

[CR8] Shammas MK, Huang X, Wu BP, Fessler E, Song I, Randolph NP, Li Y, Bleck CK, Springer DA, Fratter C et al (2022) OMA1 mediates local and global stress responses against protein misfolding in CHCHD10 mitochondrial myopathy. J Clin Invest 132:e157504 10.1172/JCI157504 PMC9282932 35700042

[CR9] Southwell N, Manzo O, Bacman S, Zhao D, Sayles NM, Dash J, Fujita K, D’Aurelio M, Di Lorenzo A, Manfredi G et al (2024) High fat diet ameliorates mitochondrial cardiomyopathy in CHCHD10 mutant mice. EMBO Mol Med. 10.1038/s44321-024-00067-5. 10.1038/s44321-024-00067-5 PMC11178915 38724625

[CR10] Wai T, García-Prieto J, Baker MJ, Merkwirth C, Benit P, Rustin P, Rupérez FJ, Barbas C, Ibañez B, Langer T (2015). Imbalanced OPA1 processing and mitochondrial fragmentation cause heart failure in mice. Science.

